# Effects of a Rehabilitation Programme with a Nasal Inspiratory Restriction Device on Exercise Capacity and Quality of Life in COPD

**DOI:** 10.3390/ijerph17103669

**Published:** 2020-05-22

**Authors:** Aurelio Arnedillo, Jose L. Gonzalez-Montesinos, Jorge R. Fernandez-Santos, Carmen Vaz-Pardal, Carolina España-Domínguez, Jesús G. Ponce-González, Magdalena Cuenca-García

**Affiliations:** 1 Pneumology, Allergy and Thoracic Surgery Department, University Hospital Puerta del Mar, 11009 Cádiz, Spain; aurelioarnedillo@neumosur.net (A.A.); caroespana@hotmail.com (C.E.-D.); 2 GALENO Research Group, Department of Physical Education, Faculty of Education Sciences, University of Cádiz, 11003 Puerto Real, Spain; jgmontesinos@uca.es (J.L.G.-M.); jorgedelrosario.fernandez@uca.es (J.R.F.-S.); magdalena.cuenca@uca.es (M.C.-G.); 3 Biomedical Research and Innovation Institute of Cádiz (INiBICA) Research Unit, Puerta del Mar University Hospital University of Cádiz, 11009 Cádiz, Spain; 4 Bahía Sur Andalusian Center for Sports Medicine, 11100 Cádiz, Spain; carmenvaz@hotmail.com; 5 MOVE-IT Research Group, Department of Physical Education, Faculty of Education Sciences, University of Cádiz, 11003 Puerto Real, Spain

**Keywords:** COPD, pulmonary rehabilitation, medical device, inspiratory muscle training

## Abstract

Objective: The objective was to assess the effects of a nasal restriction device for inspiratory muscle training, called Feelbreathe^®^, added to a rehabilitation program (RP) on exercise capacity, quality of life, dyspnea and inspiratory muscle strength in patients with stable COPD. Methods: Patients were randomized into three groups, one performed a supervised RP using the Feelbreathe^®^ device (FB group), the second group developed the same RP with oronasal breathing without FB (ONB group) and the third was the control group (CG). We evaluated inspiratory muscle strength (PImax), dyspnea (mMRC), quality of life (CAT) and exercise capacity (6MWT) before and after 8-week of RP. Results: A total of 16 patients completed the study, seven in FB group, five in ONB group and four in the CG. After the RP, the FB group showed a significant increase in PImax (93.3 ± 19.1 vs. 123.0 ± 15.8 mmHg) and in the 6MWT distance (462.9 ± 71.8 m vs. 529.1 ± 50.1 m) and a decrease in the CAT score (9.7 ± 6.5 vs. 5.9 ± 6.0) and in the mMRC dyspnea score. FB provides greater improvement in PImax, dyspnea, quality of life and 6MWT than ONB. Conclusions: The Feelbreathe^®^ device provides greater improvements in quality of life, dyspnea, exercise capacity and inspiratory muscle strength compared to patients that did not use it.

## 1. Introduction

The benefits of pulmonary rehabilitation programs (RP) in chronic obstructive pulmonary disease (COPD) patients have been shown and are recognized as an effective tool for improving dyspnea, exercise tolerance and quality of life in all the guidelines [1,2,3,4]. 

In addition to chronic airflow obstruction, COPD patients have frequent muscle dysfunction that is caused by the interaction of local and systemic factors that can involve respiratory and/or limb muscles [5,6]. Some authors explain that the limitation of COPD patients is not only in the respiratory muscles but also in the lack of skeletal muscle strength [7], which could justify carrying out respiratory muscle training and physical training jointly, so interventions that can improve the strength and endurance of the skeletal muscles, and in particular, of the inspiratory muscles, should be beneficial for these patients [8].

It remains unclear if the addition of inspiratory muscle training (IMT) to a general exercise training program leads to additional clinically relevant improvements in patients with COPD. In a review only in patients with inspiratory muscle weakness (P_Imax_ less than 60 cm H_2_O), the addition of IMT to a general exercise training program improved P_Imax_ and tended to improve exercise performance [9]. 

Current recommendations do not include IMT as an essential part of respiratory rehabilitation, however, IMT has been used as a complement added to the supervised pulmonary RP, and some studies have shown an improvement in inspiratory muscle strength, dyspnea and distance walked in the six minutes walking test (6MWT) after a IMT program in patients with COPD [10]. 

In a recent systematic review and meta-analysis, Beaumont et al. concluded that IMT using threshold devices improved inspiratory muscle strength, exercise capacity and quality of life. However, there was no added effect of IMT on dyspnea during pulmonary rehabilitation [11], but the devices used in these patients were in static position.

The methods most often used for IMT in COPD patients are resistive loading, pressure threshold loading and voluntary normocapnic hyperpnea. All these methods are currently used to improve inspiratory muscle strength [12], but have some limitations because the patients do not have physiologic respiration; must breathe by the mouth, not by the nose; and they must perform the training in static position but not in dynamic situations. 

**Figure 1:**
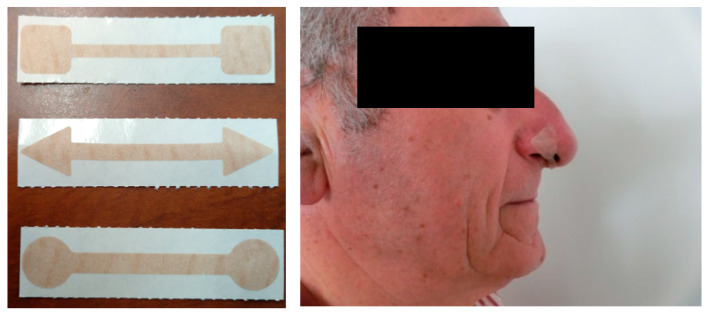
Three different sizes of the FeelBreathe^®^ device: 4, 5 and 6 mm. FeelBreathe^®^ device placed under the nostrils.

The Feelbreathe^®^ device tested in our study can be used in static and dynamic situations [13] and is a nasal ventilatory flow restriction device made by a strip of hypoallergenic material (3M Spain, S.A. Medical Specialties/O.E.M.) that is placed and adhered under the nostrils (Figure 1), impairing the free pass of air through the nose by producing resistance to flow. Depending on the size or/and porosity of the device, the inspiratory process is more or less difficult. It can be used while performing dynamic exercise or doing daily living activities. The Feelbreathe^®^ device (FB) has been authorized by the Spanish Agency for Medicines and Health Products for application on COPD patients (AEMPS-Madrid-Spain N°: U201900103, N° publication: ES1227865, 2017).

Previous studies have shown that an increased airflow resistance while breathing nasally, during exercise, increases the breathing effort [14] which may potentially improve the exercise tolerance [15] and energy efficiency [16]. In healthy subjects, FB has shown changes in lung ventilation, gas exchange and heart rate during exercise, with improvements in ventilatory efficiency [17].

Therefore, the objective of this study was to assess the effects of a nasal restriction device for inspiratory muscle training added to a supervised RP on exercise capacity, quality of life, dyspnea and inspiratory muscle strength in stable COPD patients. The hypothesis of this study was that the group who performed the training with the FB device could obtain higher benefits on exercise capacity, quality of life, dyspnea and inspiratory muscle strength.

## 2. Materials and Methods

### 2.1. Study Population

Subjects were recruited from the Pneumology outpatient of our University Hospital. Consecutive patients were screened by reviewing their charts and by interview. Inclusion criteria were diagnosis of COPD according to guidelines criteria [1,18] with moderate or severe airflow obstruction (GOLD 2 or 3) [18], dyspnea grade 2 or greater by mMRC scale and a stable clinical condition for at least 2 months. Exclusion criteria were poor compliance, treatment with oxygen therapy or non-invasive mechanical ventilation, CO_2_ retention, medical conditions that can produce or increase dyspnea on exercise in addition to COPD (cardiovascular, metabolic or other respiratory diseases) or osteoarticular or neuromuscular diseases that may limit the correct performance of the 6MWT. Participants were randomly assigned according to a computer-generated randomization table to three groups: (1) those who participated in the supervised RP using the Feelbreathe^®^ device (FB group), (2) those who participated in the supervised RP with oronasal breathing without the Feelbreathe^®^ device (ONB group) and, (3) those included in the control group (CG), which received standard medical recommendations for patients with COPD.

Written informed consent was obtained from all patients before starting the study. This clinical trial received ethical approval from the Ethics Committee University Hospital Puerta del Mar and met the requirements of the Declaration of Helsinki. ClinicalTrials.gov Identifier: NCT03936348

### 2.2. Study Protocol

**Figure 2:**
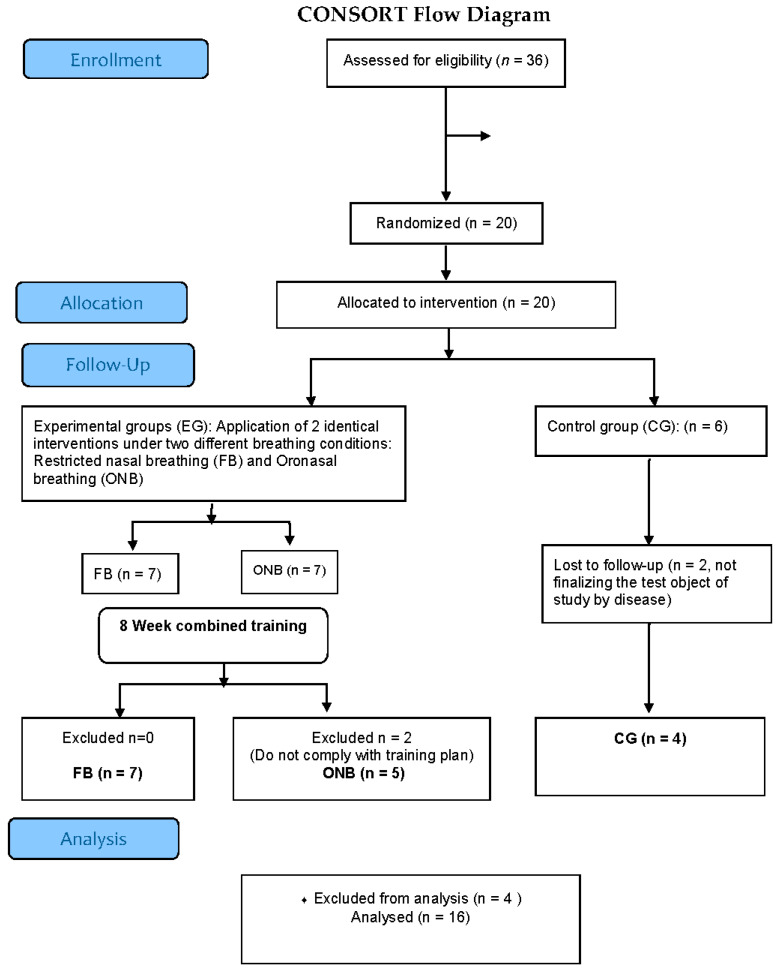
CONSORT Flow Diagram. Flow-chart showing inclusion, randomization and participation throughout the study.

A total of 20 patients were included in this study (Figure 2). Demographic and clinical data were recorded. Dyspnea was assessed by the modified Medical Research Council (mMRC) dyspnea questionnaire [19] and quality of life by the COPD Assessment Test (CAT) questionnaire [20]. Spirometry was performed according to the American Thoracic Society (ATS) criteria [21,22] (Spirometer CPX, Cardinal Health, Hoechberg, Germany). Then, they performed a resting electrocardiogram (QRS Universal ECG, QRS, Plymouth, MN, USA). P_Imax_ was measured during a maximal, static inspiratory effort measured at the mouth (Micro RPM, Micro Medical Ltd., Chatham, Kent, UK). P_Imax_ was recorded as the highest value averaged over 1s from three maneuvers that varied by less than 10% and was measured based on three maximal reproducible respiratory efforts. Then, the patients performed the 6MWT according to the ATS guideline [23].

All tests were performed according to a standardized protocol before starting the training and 2 days after its completion.

### 2.3. Training Program

Participants carried out a supervised RP for 8 weeks, 3 days per week. The pulmonary RP included therapeutic education and training sessions lasting 60 min with a warming up phase, a main phase and a recovery phase. After each session, Borg’s perceived exertion was measured [24]. The training program included aerobic exercise on cycle ergometers and on treadmills (progressing from 10′ to 30′ and from 40% to 75% of the reserve heart rate (RHR) or 6–7 score based on Borg’s perceived exertion), strengthening of lower and upper limb muscle groups, breathing exercises (pursed lip breathing, diaphragmatic and abdominal breathing and diaphragmatic mobility) and finally, stretching exercises.

In the FB group, for restricted nasal breathing, at the beginning of the training program, the small size device was used (4 mm). The size of the device was progressively increased according to the patient adaptation to the 5 or 6 mm device, depending on the score on Borg’s perceived exertion scale. If the patient had a score under 4 after the RP sessions, the size of the FB device was increased.

FB was placed under the nostrils, using sterile gloves and assessing the patient did not have mucus or injuries. The device was used during the RP and patients were encouraged to do physiological breathing by nasal inspirations and mouth expirations. 

### 2.4. Statistical Analysis

Descriptive data of the participants are expressed as mean ± standard deviation or number and percentage for continuous and categorical variables, respectively. Percentage of change (%_Change_) for each variable was calculated as:

%_Change_ = (mean(Post-test value) − mean(Pre-test value)/mean(Pre-test value)) ∗ 100 (1)

Differences among RP (between differences) and between pre- and post RP tests within each breathing condition (within differences) were analyzed using a Bayesian hierarchical model. 6MWT distance, P_Imax_ and CAT were considered as continuous variables, while mMRC dyspnea was treated as an ordinal variable. To analyze differences at baseline, only a categorical variable indicating the RP (CG, ONB or FB) was introduced in the model as predictor. However, to analyze both between and within differences RP, time (pre- or post-PR) and their interaction were introduced as predictor variables. Due to the sample size in our study, we choose to analyze our data using Bayesian inference, since it has proven to be a proper method of statistical inference for small sample size [25,26]. Weakly informative prior has been used on regression coefficients to address the issue of small sample size. This class of prior distribution encoded information to restrict the plausible range of values of a specific parameter but still leave a wide range of values to be covered [27]. Inference was performed based on the 95% credible interval (95% CrI) which contains a range of values where we can be 95% certain that the true value lies, given the data at hand and the model fitted. The Bayesian hierarchical model was fitted using the package *brms* for the R programming language for statistical computing and graphics [28]. All parameters estimated showed a good convergence with values of $\hat{R}$ = 1 and number of effective sample size >1000. The code and the dataset to replicate the analysis are stored in https://github.com/JorgeDelro/COPD_2_1.

## 3. Results

Thirty-six patients were initially screened for the study from February to March 2017. Six patients declined to participate and ten had almost one exclusion criteria. Twenty subjects were initially randomized. Two patients assigned to ONB withdrew after finishing the RP without performing the final tests, one patient in the CG withdrew for the same reason and the other one due to a COPD exacerbation. Sixteen subjects completed the study (Figure 2). 

There were no baseline differences between the groups, except in the forced vital capacity (FVC) that was worse in the ONB group compared to the CG [−665, 95% CrI (−1317, −22.9)] (Table 1). No patient was underweight or had significant hyperinflation. No patient was on oral steroid therapy or showed clinical manifestation of muscle wasting.

**Figure 3:**
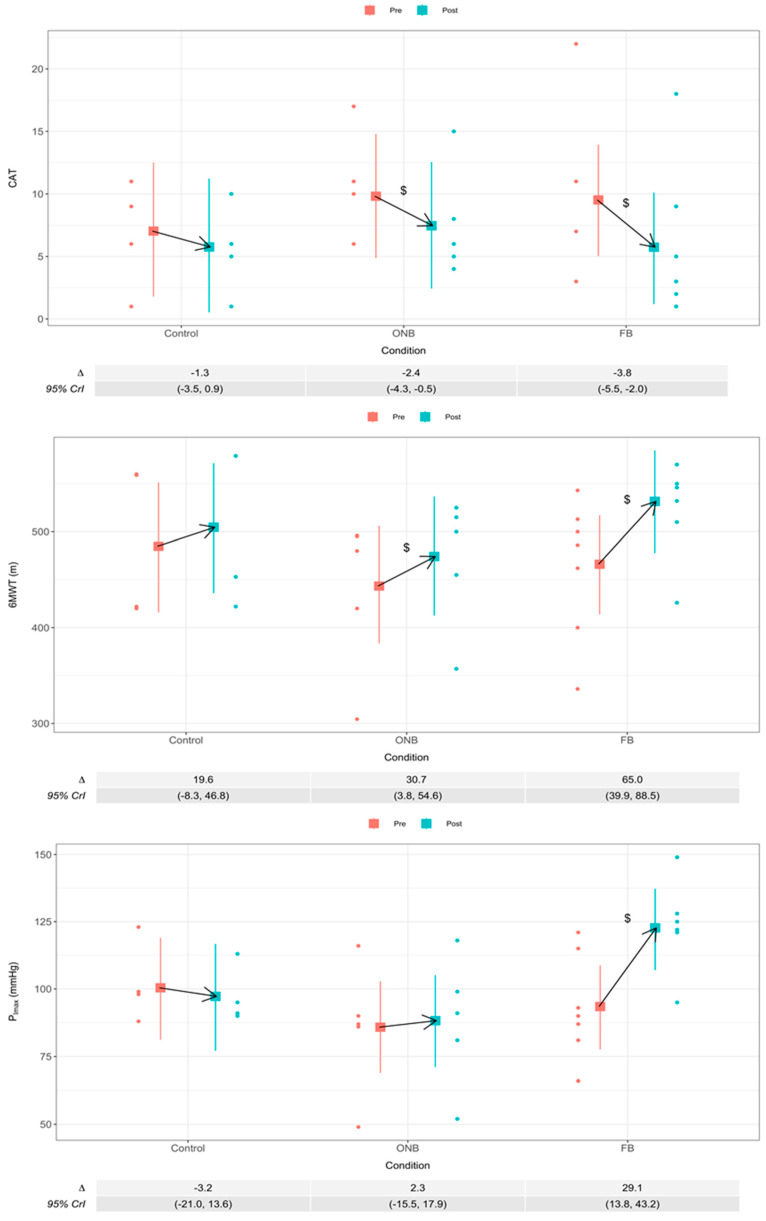
Mean difference (Δ) with 95% credible intervals (95% CrI) from pre to post-training in the CAT, 6MWT and P_Imax_. ^$^ 95% CrI does not include 0 for the difference between pre- and post-training.

The distance walked in the 6MWT increased in the groups that received PR (Figure 3). The increase was higher in the FB group than in the ONB group but both achieved the MCID defined as an increase equal or greater than 30 m [29].

**Figure 4:**
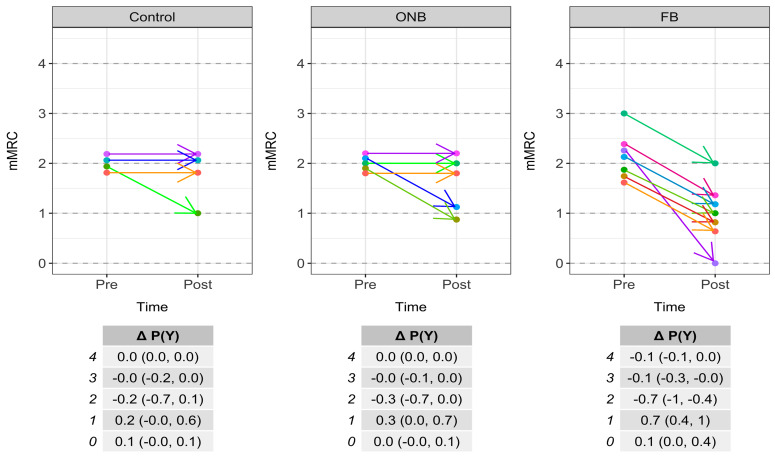
Individual changes and difference (Δ) on the probability (P) of response the row Y (i.e., 0, 1, 2, 3 or 4) from pre to post-training in the modified Medical Research Council dyspnea scale (mMRC).

Comparing changes in dyspnea, the probability of achieving a reduction in 1 point in the mMRC dyspnea scale, which is considered as MCID, was greater than 0.5 in the FB group and less than 0.5 in the ONB and CG (Figure 4).

In the FB and ONB groups, the quality of life measure by CAT questionnaire improves, with the improvement greater in the FB group compared to the ONB group (−3.8 vs. −2.4, respectively) (Figure 3). In both groups, the CAT score decreases more than 2 points achieving the MCID [30]. In the CG, there were no significant changes in CAT score (Figure 3).

P_Imax_ improved significantly in the FB group, without significant changes in the ONB and CG (Figure 3).

Table 2 shows differences comparing the three groups after and before pulmonary RP. The FB group achieves a significant increase in the P_Imax_ compared to the ONB group, and in the P_Imax_ and 6MWT compare to the CG.

## 4. Discussion

In this prospective randomized trial, the Feelbreathe^®^ device added on a supervised pulmonary RP in stable COPD patients provides greater improvements in quality of life, dyspnea, exercise capacity and inspiratory muscle strength compared to patients that did not use the FB device. 

The 6MWT has been used to assess the efficacy of pulmonary RP, and the vast majority of these studies have shown an improvement in the distance walked in 6 min [31]. Due to sometimes having statistical significant differences that may not reflect a clinically relevant change, Jaeschke et al. defined the term minimal clinically important difference (MCID) as “the smallest difference in score in the domain of interest which patients perceive as beneficial” [32]. For pulmonary RP, an improvement in at least 30 m in the 6MWT is considered as MCID [29,33] and in this study, the FB and ONB groups achieved MCID, but the improvements were greater, more than double, in the FB group.

The concept of MCID can be applied to the CAT questionnaire. In this case, a change of at least 2 points in the score of the CAT is considered as MCID [30]. In this study, the FB and ONB groups achieved the MCID. The decrease in the CAT score was higher in the FB group, which may reflect the effect of the better exercise capacity of the patients [34], however, the difference between both groups did not achieve the MCID.

Only the FB group showed a significant decrease in the mMRC dyspnea score. Other studies have described improvements in dyspnea after a RP with exercise training in COPD patients, but usually with programs with high intensity (70%–80% maximum workload) [35,36], but in this study, the intensity of the exercise training was close to 60%, ranging from 40 to 75%. 

Finally, only the FB group increased significantly the P_Imax_. This may be due to the FB device being able to provide a greater effect on inspiratory muscles, because it trains the inspiratory muscles while performing the whole rehabilitation session, unlike the others devices currently available for IMT that only can be used in static situation [17]. 

Lötters F et al., in a meta-analysis comparing IMT alone and IMT as adjunct to general RP in patients with COPD, demonstrated a significant increase in the inspiratory muscle strength and endurance in both groups, especially in patients with inspiratory muscle weakness (P_Imax_ < 60 cm H_2_O) [10]. In those studies, when the patients have a ventilatory limitation during exercise, the addition of IMT to a RP increased the exercise capacity. This finding has been described by other authors [37,38]. 

Magadle et al. assessed the influence of adding IMT to patients with COPD after finishing a 12 week RP [39]. IMT group showed a significant increase in P_Imax_ and quality of life and decrease in the Borg scale dyspnea score, remaining unchanged the 6MWT, unlike our study in which FB and ONG groups improved the 6MWT distance. They argued that the absence of an additive influence of IMT to a pulmonary RP could be because the patients had achieved their full potential in response to pulmonary RP alone. In our study, the FB and the ONB groups performed a complete pulmonary RP that is highly effective for patients with COPD and further improvements in exercise capacity adding on other interventions may be challenging to obtain, but this is an excellent way to better discriminate the effect of the IMT in both groups.

Beaumont et al. [40] compared two PR programs, one with IMT versus no IMT in 32 COPD patients without inspiratory muscle weakness (P_Imax_ > 60 cm H_2_O) as in our study, to demonstrate the effectiveness of IMT on dyspnea, P_Imax_ and 6MWT. The RP was very similar to ours, but the duration was only three weeks. They found a trend toward an improvement in dyspnea that was statistically significant in patients with FEV_1_ less than 50%, but no changes were observed in 6MWT and P_Imax_.

Finally, in a recent systematic review, Camillo et al. [41] analyzed the effectiveness of therapies added on to conventional exercise training to maximize exercise capacity in patients with COPD. When IMT was used as add-on therapy to the RP, there was an improvement in the 6MWT distance in favor to the experimental group (12.72 m (95%CI −16.81–42.26)), but this increase was discrete and below the MCID.

The most important difference between the studies analyzed and our study is the use of a new inspiratory restriction device (Feelbreathe^®^), that allows an IMT during all the period that the patient is receiving the pulmonary RP, so the action of the intervention on inspiratory muscles is continuous and probably of greater intensity. This device allows for a simultaneous training of the respiratory and skeletal muscles when the patients are receiving the rehabilitation. The IMT performed in the rest of the studies is in static situations and the action of the IMT is for a limited period of time during the whole pulmonary RP. 

In our study, the baseline inspiratory muscle pressure was over 60 mmHg in all groups, nonetheless, the FB group showed improvements in the final P_Imax_, and this may be due to the same reason, a higher intensity in the IMT developed by the FB device.

A limitation of our study was the sample size and the high number of drop off leading to small size unbalance pulmonary rehabilitation groups. To address this problem, we analyzed our data relying on the principles of Bayesian hierarchical modeling (i.e., quantify the uncertainty around a quantity based on the data we have at hand using prior information about that quantity). Moreover, the hierarchical part of the modeling (also known as multilevel modeling) allowed us to obtain a reliable estimate of the parameters by partial pooling. 

Another limitation of our study is that the design was prospective randomized but open, because it was impossible to perform a double-blind design due to the characteristics of the device.

As one of the advantages of the FB device is that it is easy to use and the possibility of being used safely in dynamic situations, so it could be beneficial for training the inspiratory muscles in ambulatory patients with COPD, for example, while they are walking or during their daily activities or to maintain the long-term effects of a supervised pulmonary RP.

## 5. Conclusions

In conclusion, the Feelbreathe^®^ device can be useful as an add-on therapy to a supervised pulmonary rehabilitation program for patients with COPD, improving dyspnea, quality of life, exercise tolerance and inspiratory muscle strength. According to these results, in the future, it could be interesting to study if the Feelbreathe^®^ device could be useful for unsupervised exercise training or for daily activities in patients with COPD.

## Figures and Tables

**Table 1:** Baseline characteristics of the patients.

Variables	FB (*n* = 7)	ONB (*n* = 5)	CG (*n* = 4)	FB vs. ONB	FB vs. CG	ONB vs. CG
Age (years)	65 ± 8.0	72 ± 7.4	70.2 ± 25.9	−4.8 (−14.8, 6.2)	−6.6 (−15.5, 3.6)	1.7 (−10.0, 12.9)
BMI (m/kg^2^)	28.4 ± 4.2	26.8 ± 2.5	25.9 ± 2.1	1.5 (−2.4, 5.4)	2.3 (−2.0, 6.3)	0.8 (−3.6, 5.5)
DLCO (%)	72.3 ± 20.0	71.4 ± 8.8	75.2 ± 16.4	0.85 (-9.8, 11.5)	−2.95 (−13.5, 10.5)	−3.8 (−15.8, 8.2)
RV/TLC (%)	86.0 ± 10.6	84.5 ± 12.0	85.8 ± 14.6	1.5 (-6.4, 9.4)	0.3 (−7.6, 8,1)	1.3 (−7.4, 9.9)
FEV (mL)	1571 ± 334	1608 ± 344	1812 ± 706	−23.5 (−557, 469)	−218 (−760, 349)	194 (−441, 773)
FEV (%)	46.9 ± 10.6	51.2 ± 9.8	52.6 ± 19.9	−3.7 (−18.4, 11.1)	−5.4 (−21.6, 10.8)	−1.8 (−19.8, 16.1)
FVC (mL)	2869 ± 298	2580 ± 577	3270 ± 474	283 (−260, 801)	−382 (−929, 207)	−665 (−1317, −22.9) ^$^
FVC (%)	63.9 ± 8.3	59.2 ± 10.0	67.1 ± 13.8	4.5 (−7.4, 17.1)	−3.1 (−15.9, 10.1)	−7.6 (21.5, 7.7)
FEV/FVC (%)	54.1 ± 6.9	62.6 ± 5.6	54.2 ± 14.7	−8.1 (−18.7, 2.8)	0.0 (−11.4, 11.4)	8.1 (−4.2, 20.8)
P_Imax_ (mmHg)	93.3 ± 19.1	85.6 ± 23.9	102 ±14.85	7.9 (−15.6, 31.1)	−7.6(−34.3, 17.3)	−15.5 (−42.8, 12.5)
6MWT (m)	462.9 ± 71.8	439.1 ± 81.4	490.3 ± 80.0	23.6 (−68.6, 108.0)	−25.8 (−115.0, 75.1)	−49.4 (−148.0, 48.7)
CAT score	9.7 ± 6.5	10.0 ± 4.5	6.8 ± 4.4	−0.3 (−6.8, 6.4)	2.7 (−3.8, 9.7)	3.0 (−4.5, 10)
mMRC (score/%)				P(Y|FB)	P(Y|ONB)	P(Y|CG)
0	0 (0%)	0 (0%)	0 (0%)	0.0 (0.0–0.0)	0.0 (0.0–0.0)	0.0 (0.0–0.0)
1	0 (0%)	0 (0%)	0 (0%)	0.0 (0.0–0.1)	0.0 (0.0–0.2)	0.0 (0.0–0.2)
2	6 (85%)	5 (100%)	4 (100%)	0.9 (0.6–1) ^$^	0.9 (0.7–1) ^$^	0.9 (0.7–1) ^$^
3	1 (15%)	0 (0%)	0 (0%)	0.1 (0.0–0.4)	0.0 (0.0–0.2)	0.0 (0.0–0.3)

BMI: body mass index; CAT: COPD Assessment Test (range of zero to 40); DLCO: diffusing capacity of the lung for carbon monoxide; RV/TLC: residual volume/total lung capacity; FEV1, forced expiratory volume in the first second; FVC, forced vital capacity; PI_max_, maximal inspiratory pressure; 6MWT, 6 min walking test; mMRC: modified Medical Research Council dyspnea scale. CG: control group, FB: Feelbreathe group. ONB: oronasal breathe group. FB vs. ONB/FB vs. CG/ONB vs. CG: mean difference and 95% credible interval between groups. P(Y|FB)/P(Y|ONB)/P(Y|CG): probability of answer the score Y (i.e., 0, 1, 2, 3), given a participant was assigned to a group at baseline. ^$^ 95% credible interval does not include 0 for continuous variables or 0.5 for categorical variables.

**Table 2:** Between-differences for breathing conditions after and before pulmonary rehabilitation.

Variables	FB vs. ONB	FB vs. CG	ONB vs. CG
CAT	−1.4 (−3.8–1.3)	−2.5 (−5.2–0.5)	−1.1 (−3.7–2.0)
6MWT (m)	34.2 (−0.2–70.7)	45.4 (10.3–81.6) ^$^	11.2 (−23.1–49.0)
P_Imax_ (mmHg)	26.8 (3.7–49.4) ^$^	32.3 (10.5–55.4) ^$^	5.5 (−17.5–29.2)

FB: Feelbreathe group. ONB: oronasal breathe group. CG: control group. CAT: COPD Assessment Test; 6MWT, 6 min walking test; PI_max_, maximal inspiratory pressure. ^$^ 95% credible interval does not include 0.
